# The fluid challenge

**DOI:** 10.1186/s13054-020-03443-y

**Published:** 2020-12-28

**Authors:** Jean-Louis Vincent, Maurizio Cecconi, Daniel De Backer

**Affiliations:** 1 Department of Intensive Care, Erasme Hospital, Université Libre de Bruxelles, Route de Lennik 808, 1070 Brussels, Belgium; 2 Department of Anesthesiology and Intensive Care, Humanitas Clinical and Research Center-IRCCS, Rozzano, Milan Italy; 3 Department of Biomedical Sciences, Humanitas University, Pieve Emanuele, Milan Italy; 4 Department of Intensive Care, CHIREC Hospital, Université Libre de Bruxelles, Brussels, Belgium

The primary goal of fluid administration is to increase cardiac output and therefore oxygen delivery by the Frank–Starling relationship, which relates stroke volume (or cardiac output) to a cardiac filling volume (Fig. 1). However, if there is no concurrent fluid loss (for example in hemorrhage), fluid administration can result in an increase in hydrostatic pressures with ensuing edema formation. Therefore, fluid administration can be associated with a potential benefit (increase in cardiac output) and a risk of harm (increase in hydrostatic pressure). Different patients, and the same patient at different times during their illness, will have different requirements to increase their oxygen delivery and will be on different parts of the Frank–Starling curve.

**Fig. 1:**
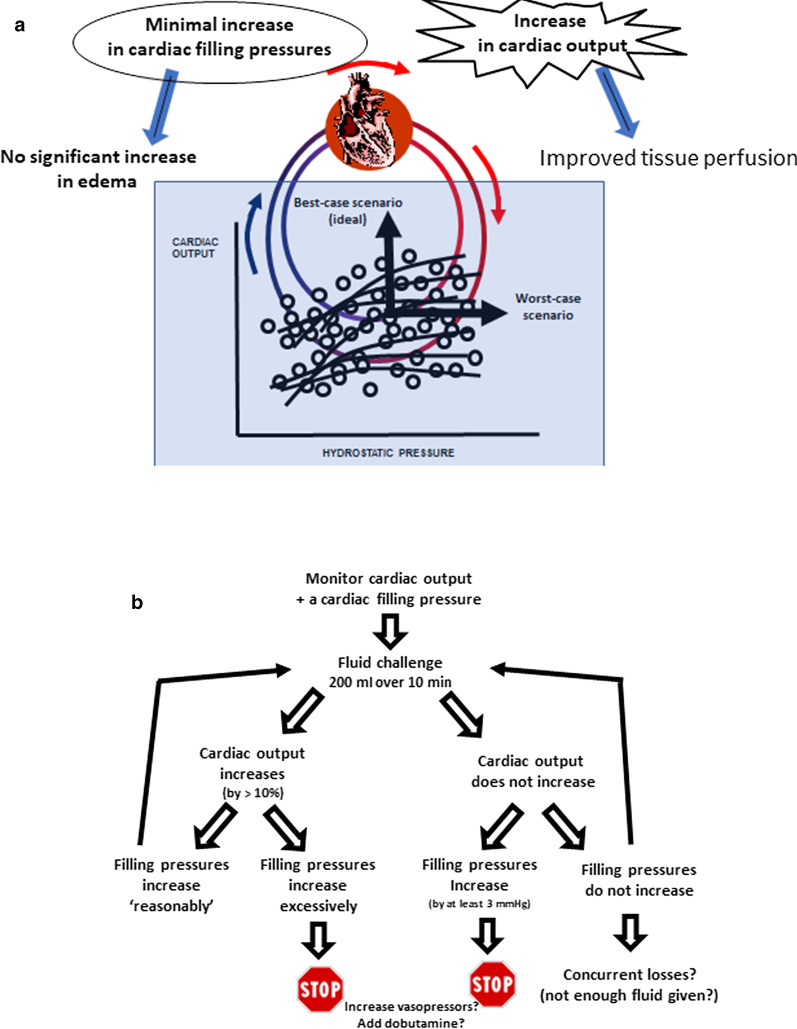
**a** The concept underlying the fluid challenge technique. The best-case scenario for the tissues would be a significant increase in cardiac output with a minimal increase in cardiac filling pressures, whereas the worst-case scenario is a major increase in cardiac filling pressures with no significant increase in cardiac output. **b** The practical approach to a fluid challenge

A fluid challenge is the safest way to administer fluids when we consider they may be beneficial but are unsure they will be well tolerated. As initially described by Weil and Henning [1], the principle of the fluid challenge technique is to administer a bolus of intravenous fluid under tightly controlled conditions and to evaluate the patient’s hemodynamic response. The fluid challenge technique thus evaluates the balance between the benefit—increase in oxygen delivery to the tissues—and the risk—increased edema formation [2]. If there is no clinical benefit (i.e., no increase in cardiac output), fluid administration should be rapidly interrupted. If there is a modest increase in cardiac output, the degree of concurrent increase in cardiac filling pressure (often estimated using the central venous pressure [CVP]) should be estimated. If the cardiac filling pressures are estimated to be low, the risk of edema formation is limited, so that a strict fluid protocol may not be necessary. In other conditions, where lung function can deteriorate (e.g., acute respiratory distress syndrome [ARDS] or cardiogenic shock), a fluid challenge protocol becomes indispensable.

Importantly, any given CVP value will not accurately predict whether or not a patient will respond to fluids; this is true for all variables, including the cardiac output, capillary refill time, central venous oxygen saturation (ScvO_2_), urine output or blood lactate level. Nevertheless, when the CVP is low there are greater chances of an increase in cardiac output in response to fluids. This was illustrated in a small study in which more than 80% of patients with a CVP ≤ 5 mmHg responded to fluids, but perhaps more importantly between 40 and 50% of patients with a high CVP still responded [3].

It is important to perform a fluid challenge properly, to maximize the positive and negative predictive values. Two essential components are the amount of fluid administered and the duration over which it is given. Too little fluid may not result in any significant hemodynamic change, but a large amount of fluid may result in a positive response in any individual. Fujimoto et al. [4] showed in healthy volunteers that a fluid bolus of about 1 L of saline over 5–10 min resulted in an increase in cardiac index from 3.2 to 4.0 L/min/m^2^. In critically ill patients, the optimal volume seems to be around 4 ml/kg [5]. The duration is even more important: many things can happen during a fluid challenge that may influence the result, including changes in position and therapies, among others, so that a fluid challenge over a 20 or 30 min time period would not make sense and a shorter period of 5–10 min during which all other factors can be kept unchanged is recommended [2, 6]. A mini-fluid challenge, in which 100 ml of fluid is given over just 1 min, has been proposed in the operating room, where patient status can change very quickly [7]. The method used to monitor cardiac output does not matter much, as long as it is reliable. The maximal change in cardiac output should be assessed 1 min after the end of the fluid infusion [8]. The least invasive technique that could be used is measurement of the velocity time integral using the Doppler technique and averaging three measurements by the same operator [9].

The initial description proposed strict rules for stopping a fluid challenge [1], but we feel the need to continue or to stop should be individualized with a clearly defined objective and limit for each patient, so that the best balance of benefit vs. potential harm can be determined (Fig. 1). For a fluid challenge to be considered positive, a sufficient increase in cardiac output will be necessary, with a 10% increase generally considered as a minimum using our current measurement techniques. At the same time, we should carefully monitor a cardiac filling pressure (usually the CVP) to make sure it does not increase dangerously. In all cases, if there is no increase in cardiac output and cardiac filling pressures increase, the fluid challenge should be promptly discontinued.

The fluid challenge may have to be repeated to assess ongoing fluid requirements. But how often should this be done? Too often carries the risks associated with giving too much fluid, and not often enough may prevent the patient receiving sufficient fluid. In a French multicenter trial, Roger et al. [10] observed that the positive effects of a fluid challenge were transient in 40% of patients. Whether or not the fluid challenge should then be repeated should be carefully evaluated by the clinician. Importantly, any fluid challenge should be considered a physiological experiment and its effects carefully evaluated. Unfortunately, in too many cases, further fluid is administered independent of the response to a fluid challenge; this is not good practice.

In conclusion, one should remember that use of a fluid challenge technique will result in not more but less fluid being given in total, because fluid administration will be quickly discontinued if there is no clinical benefit. The fluid challenge technique should be adapted to the individual patient, with each component defined in advance according to the TROL mnemonic: Type of fluid (usually a crystalloid), Rate of infusion (typically 200 ml over about 10 min), Objective (usually an increase in cardiac output by at least 10%) and Limits (excessive increase in CVP).

## Data Availability

Not applicable.

## Abbreviations

ARDS: Acute respiratory distress syndrome
CVP: Central venous pressure
PAOP: Pulmonary artery occlusion pressure
ScvO_2_: Central venous oxygen saturation
